# Spike-antibody responses to COVID-19 vaccination by demographic and clinical factors in a prospective community cohort study

**DOI:** 10.1038/s41467-022-33550-z

**Published:** 2022-10-02

**Authors:** Madhumita Shrotri, Ellen Fragaszy, Vincent Nguyen, Annalan M. D. Navaratnam, Cyril Geismar, Sarah Beale, Jana Kovar, Thomas E. Byrne, Wing Lam Erica Fong, Parth Patel, Anna Aryee, Isobel Braithwaite, Anne M. Johnson, Alison Rodger, Andrew C. Hayward, Robert W. Aldridge

**Affiliations:** 1 Institute of Health Informatics, University College London, London, UK; 2 Department of Infectious Disease Epidemiology, London School of Hygiene and Tropical Medicine, Keppel Street, London, UK; 3 Institute of Epidemiology and Health Care, University College London, London, UK; 4 Institute for Global Health, University College London, London, UK

**Keywords:** Policy and public health in microbiology, Vaccines, SARS-CoV-2, Antibodies

## Abstract

Vaccination constitutes the best long-term solution against Coronavirus Disease-2019; however, vaccine-derived immunity may not protect all groups equally, and the durability of protective antibodies may be short. We evaluate Spike-antibody responses following BNT162b2 or ChAdOx1-S vaccination amongst SARS-CoV2-naive adults across England and Wales enrolled in a prospective cohort study (Virus Watch). Here we show BNT162b2 recipients achieved higher peak antibody levels after two doses; however, both groups experience substantial antibody waning over time. In 8356 individuals submitting a sample ≥28 days after Dose 2, we observe significantly reduced Spike-antibody levels following two doses amongst individuals reporting conditions and therapies that cause immunosuppression. After adjusting for these, several common chronic conditions also appear to attenuate the antibody response. These findings suggest the need to continue prioritising vulnerable groups, who have been vaccinated earliest and have the most attenuated antibody responses, for future boosters.

## Abstract (web summary)

Vaccination can provide reliable and long-lasting protection against COVID-19, however the immune response to vaccination can vary between individuals and can decline over time, leading to differences in protective effects. Here the authors assess the immune response to COVID-19 vaccination across a large cohort of previously uninfected adults and demonstrate lower post-vaccination antibody levels amongst those with immune-suppressing conditions and medications, as well as those with several other more common chronic conditions.

## Introduction

The ongoing Severe Acute Respiratory Syndrome Coronavirus 2 (SARS-CoV-2) pandemic and the morbidity and mortality resulting from the associated clinical syndrome, Coronavirus disease-2019 (COVID-19), has had a devastating impact on many nations with 565,207,160 cumulative cases and 6,373,739 deaths reported globally as of 22 July 2022^1^. Early in the pandemic, global vaccine developers joined the race to find a definitive, long-term solution, pivoting both established and experimental vaccine platforms towards SARS-CoV-2. Pfizer/BioNTech’s BNT162b2 messenger RNA vaccine, and Oxford/AstraZeneca’s ChAdOx1-S non-replicating adenovirus-vectored vaccine, based on the Spike protein of wild-type SARS-CoV-2, were licensed in the UK in December 2020 and have been rolled out in England as homologous prime-boost regimens, with an 8–12 weeks dose interval^2^. The Spike-based formulation of these vaccines allows antibodies against the Nucleocapsid protein, an abundant and highly immunogenic viral antigen^3^, to remain discriminatory for natural infection. Vaccination has been offered in accordance with the UK’s Joint Committee on Vaccination and Immunisation’s clinical prioritisation framework^4^, with 45,134,956 people (93.3% of the UK population aged 12 and over) having received their first vaccine dose, and 42,347,786 (87.5%) having received the full primary course of two doses as of 19 July 2022, and with booster (third and fourth doses) campaigns now also well underway^5^.

Trial and observational data have demonstrated the efficacy of ChAdOx1 and BNT162b2 vaccines against infection and clinical severity^6–10^; however, while there are abundant trial data on immunogenicity, this evidence is generally limited to younger, healthier trial populations; and observational data from countries other than the U.K. is generally limited to manufacturer-recommended dosing intervals of 3–4 weeks or to the Pfizer vaccine. In the UK, the recommended dosing interval was extended to 8–12 weeks in order to maximise first-dose coverage across the population^2^, with subsequent data also suggesting that longer dosing intervals lead to higher immunogencity^11,12^. Another significant feature of the U.K vaccination programme is that clinically vulnerable groups, including those with common chronic conditions as well as those with more significant immuno-suppressive conditions or therapies, have been prioritised for vaccination^4^. There is accumulating data suggesting reduced immune responses to first and second-dose vaccination in specific highly vulnerable groups, such as solid-organ transplant recipients^13–15^, those on powerful disease-modifying immunosuppressants or immune-modulators^16–18^, and those with haematological malignancies^19,20^, however most studies have focused on these rare clinical groups in isolation, without including a broader range of common clinical conditions, such as diabetes, heart disease, and obesity, and few have adjusted for demographic factors, such as age, sex, and ethnicity, or for vaccination factors such as type, dose interval, and time since vaccination.

There remains a need to understand differences in humoral immune responses across clinical groups following longer dosing intervals, and receipt of the Oxford-AstraZeneca vaccine; particularly amongst older people, those with common metabolic risk factors, and those from ethnic minority backgrounds, as these groups are at highest risk of COVID-19 morbidity and mortality^21–24^; as well as those on a broader range of immunosuppressive therapies that could attenuate vaccine responses^25^. It is particularly important to assess disparities in humoral responses in a robust way, by adjusting for key confounders, to inform long-term vaccination strategies across the population.

Here we analyse serological data from a large, prospective community cohort across England and Wales (Virus Watch)^26^, obtained using a widely available, validated commercial assay. We use multivariable linear regression to show disparities in Spike-antibody levels following two vaccine doses in individuals without evidence of prior infection, with a range of common and rare clinical risk factors.

## Results

### S-antibody levels over time

Of the 44,204 individuals enrolled in Virus Watch, a total of 9320 individuals, who submitted samples between 24 February 2021 and 13 October 2021, were eligible for inclusion in the descriptive analysis of antibody levels over time following Dose 1 (Table 1, Supplementary Table S1, Fig. S1a). For the corresponding Dose 2 analysis, 8471 individuals who submitted samples between 1 July 2021 and 17 December 2021 were included (Table 1, Supplementary Fig. S1b). For these descriptive analyses, individuals contributed a median of 5 (IQR 2, 9) samples each. Amongst included ChAdOx1-S recipients the proportions of individuals aged 45–64 years and ≥65 years were roughly equal, while BNT162b2 recipients were predominantly ≥65 years. The proportions of males and females, and White British and Minority Ethnic groups, and the prevalence of most clinical risk factors of interest were roughly equal across the two vaccine types for both Dose 1 and 2 cohorts.

**Table 1:** Demographic and clinical characteristics of individuals who were sampled following Dose 1 and Dose 2 of either ChAdOx1-S or BNT162b2 vaccines

	Dose 1 cohort (day ≥0)		Dose 2 cohort (day ≥0)	
Vaccine Type	ChAdOx1 *N* = 5964	BNT162b2 *N* = 3266	ChAdOx1 *N* = 5610	BNT162b2, *N* = 2861
Age group (years)				
18–24	25 (0.4%)	91 (2.8%)	21 (0.4%)	54 (1.9%)
25–44	548 (9.2%)	454 (14%)	444 (7.9%)	326 (11%)
45–64	2868 (48%)	810 (25%)	2775 (49%)	817 (29%)
65+	2523 (42%)	1911 (59%)	2370 (42%)	1664 (58%)
Sex				
Male	2551 (43%)	1420 (43%)	2375 (42%)	1221 (43%)
Female	3413 (57%)	1846 (57%)	3235 (58%)	1640 (57%)
Ethnicity				
White British	5523 (93%)	2943 (90%)	5212 (93%)	2589 (90%)
Minority ethnic	441 (7.4%)	323 (9.9%)	398 (7.1%)	272 (9.5%)
BMI				
Normal	2290 (38%)	1237 (38%)	2003 (36%)	1021 (36%)
Obese	1183 (20%)	614 (19%)	1009 (18%)	461 (16%)
Overweight	1982 (33%)	1112 (34%)	1741 (31%)	899 (31%)
Underweight	71 (1.2%)	35 (1.1%)	51 (0.9%)	34 (1.2%)
Missing	438 (7.3%)	268 (8.2%)	806 (14%)	446 (16%)
Clinical vulnerability (JCVI)				
Not clinically vulnerable	3439 (58%)	1693 (52%)	3411 (61%)	1588 (56%)
Clinically vulnerable	1762 (30%)	1061 (32%)	1501 (27%)	823 (29%)
Clinically extremely vulnerable	763 (13%)	512 (16%)	698 (12%)	450 (16%)
Solid organ cancer (non-Haem)	484 (8.1%)	311 (9.5%)	412 (7.3%)	254 (8.9%)
Haematological cancer	55 (0.9%)	31 (0.9%)	48 (0.9%)	32 (1.1%)
Haematological non-malignant condition	43 (0.7%)	23 (0.7%)	33 (0.6%)	19 (0.7%)
Asthma	1013 (17%)	549 (17%)	912 (16%)	472 (16%)
COPD	175 (2.9%)	127 (3.9%)	147 (2.6%)	97 (3.4%)
Severe respiratory disease	320 (5.4%)	160 (4.9%)	290 (5.2%)	147 (5.1%)
Ischaemic heart disease	265 (4.4%)	205 (6.3%)	223 (4.0%)	151 (5.3%)
Hypertension	1561 (26%)	1028 (31%)	1,378 (25%)	833 (29%)
Heart failure	28 (0.5%)	24 (0.7%)	23 (0.4%)	17 (0.6%)
Chronic viral (HBV, HCV, HIV)	31 (0.5%)	9 (0.3%)	33 (0.6%)	12 (0.4%)
Type 1 DM	29 (0.5%)	17 (0.5%)	29 (0.5%)	18 (0.6%)
Type 2 DM	264 (4.4%)	195 (6.0%)	237 (4.2%)	174 (6.1%)
Stroke	98 (1.6%)	70 (2.1%)	76 (1.4%)	55 (1.9%)
Neurological condition (excl. stroke)	122 (2.0%)	82 (2.5%)	110 (2.0%)	58 (2.0%)
Mental ill-health	90 (1.5%)	50 (1.5%)	84 (1.5%)	35 (1.2%)
Liver condition	113 (1.9%)	64 (2.0%)	101 (1.8%)	44 (1.5%)
Inflammatory conditions	372 (6.2%)	256 (7.8%)	367 (6.5%)	245 (8.6%)
Chronic Kidney Disease	68 (1.1%)	59 (1.8%)	55 (1.0%)	46 (1.6%)
Immunosuppressive Drugs				
Steroids (long course)	91 (1.5%)	44 (1.3%)	77 (1.4%)	37 (1.3%)
DMARDs	111 (1.9%)	85 (2.6%)	116 (2.1%)	79 (2.8%)
MABs	39 (0.7%)	34 (1.0%)	39 (0.7%)	29 (1.0%)
Samples available from day ≥28	5530 (92.7%)	2984 (91.4%)	5568 (99.3%))	2788 (97.4%)

**Fig. 1:**
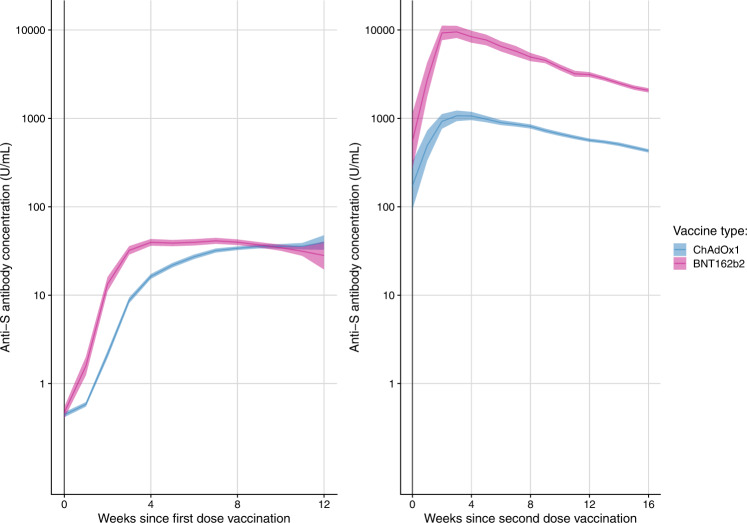
S-antibody levels over time following the first dose (left) and the second dose (right) of COVID-19 vaccination. Geometric means and 95% confidence intervals of Spike-antibody (U/ml) for each week since receiving first and second doses of vaccination, stratified by vaccine type (blue = ChAdOx1-S; pink = BNT162b2).

On plotting Dose 1 samples by time since vaccination, it was evident that S-antibody levels for BNT162b2 recipients rose more quickly than ChAdOx1-S recipients, however both groups achieved similar levels by eight weeks after vaccination (Fig. 1). At 12 weeks after vaccination, there is a suggestion of slight waning in levels for BNT162b2 recipients, whilst levels in ChAdOx1-S recipients continue to rise very slightly.

Following Dose 2, S-antibody levels once again rose more sharply for BNT162b2 recipients, this time achieving substantially higher peak levels than ChAdOx1 recipients. For both groups, antibody levels peaked around 2–4 weeks after vaccination, and then appeared to decline at a similar log-linear rate between 4 and 16 weeks after vaccination. Peak antibody levels following Dose 2 were remarkably higher than peak levels after Dose 1, and remained so even at 16 weeks after Dose 2.

### S-antibody seroconversion

A total of 8514 individuals submitted a serum sample that was taken ≥28 days following Dose 1 and prior to Dose 2. Taking the earliest valid sample within this timeframe from each participant, 166/5364 of ChAdOx1 recipients and 86/2898 of BNT162b2 recipients remained seronegative at ≥28 days following Dose 1, with *n* = 57 (34% [27, 42]) and *n* = 42 (49% [38, 60]) of these individuals classified as Clinically Extremely Vulnerable, respectively (Table 2). For both ChAdOx1-S and BNT162b2 recipients respectively, use of long steroid courses (*n* = 16, 9.6% [5.8, 15]; *n* = 7, 8.1% [3.6, 17]), DMARDs (*n* = 23, 14% [9.2, 20]; *n* = 15, 17% [10, 27]), MABs (*n* = 13, 7.8% [4.4, 13]; *n* = 5, 5.8% [2.2, 14]), and diagnosis of haematological malignancy (*n* = 15, 9.0% [5.3, 15]; *n* = 7, 8.1% [3.6, 17]) appeared highly over-represented in the seronegative category, compared to the wider cohort and the seropositive category. Obesity and diagnoses of ischaemic heart disease, solid organ cancer, Type 1 and 2 diabetes mellitus, heart failure, and neurological, liver, and inflammatory conditions also appeared to be slightly over-represented in the seronegative groups, for both ChAdOx1-S and BNT162b2 respectively.

**Table 2:** Demographic and clinical characteristics of individuals who were seronegative and individuals who were seropositive (as per the ≥0.8 U/ml cut-off for Spike-antibody level) at ≥28 days after receiving Dose 1, with percentage estimates and 95% confidence intervals for seropositivity and seronegativity rates

	ChAdOx1 Dose 1 (≥28 days)				BNT162b2 Dose 1 (≥28 days)			
	Seronegative *N* = 166		Seropositive *N* = 5364		Seronegative *N* = 86		Seropositive *N* = 2898	
	*n* (%)	95% CI^a^	*n* (%)	95% CI^a^	*n* (%)	95% CI^a^	*n* (%)	95% CI^a^
Age group (years)								
18–24	0 (0%)	0.00%, 2.8%	20 (0.4%)	0.23%, 0.59%	1 (1.2%)	0.06%, 7.2%	61 (2.1%)	1.6%, 2.7%
25–44	10 (6.0%)	3.1%, 11%	454 (8.5%)	7.7%, 9.2%	7 (8.1%)	3.6%, 17%	303 (10%)	9.4%, 12%
45–64	63 (38%)	31%, 46%	2545 (47%)	46%, 49%	17 (20%)	12%, 30%	736 (25%)	24%, 27%
65+	93 (56%)	48%, 64%	2345 (44%)	42%, 45%	61 (71%)	60%, 80%	1798 (62%)	60%, 64%
Sex								
Male	95 (57%)	49%, 65%	2268 (42%)	41%, 44%	47 (55%)	44%, 65%	1254 (43%)	41%, 45%
Female	71 (43%)	35%, 51%	3096 (58%)	56%, 59%	39 (45%)	35%, 56%	1644 (57%)	55%, 59%
Ethnicity								
White British	153 (92%)	87%, 96%	4985 (93%)	92%, 94%	77 (90%)	81%, 95%	2646 (91%)	90%, 92%
Minority ethnic	13 (7.8%)	4.4%, 13%	379 (7.1%)	6.4%, 7.8%	9 (10%)	5.2%, 19%	252 (8.7%)	7.7%, 9.8%
BMI								
Normal	62 (37%)	30%, 45%	2064 (38%)	37%, 40%	35 (41%)	30%, 52%	1096 (38%)	36%, 40%
Obese	38 (23%)	17%, 30%	1043 (19%)	18%, 21%	22 (26%)	17%, 36%	535 (18%)	17%, 20%
Overweight	53 (32%)	25%, 40%	1801 (34%)	32%, 35%	24 (28%)	19%, 39%	1024 (35%)	34%, 37%
Underweight	0 (0%)	0.00%, 2.8%	68 (1.3%)	1.0%, 1.6%	2 (2.3%)	0.40%, 8.9%	32 (1.1%)	0.77%, 1.6%
Missing	13 (7.8%)	4.4%, 13%	388 (7.2%)	6.6%, 8.0%	3 (3.5%)	0.91%, 11%	211 (7.3%)	6.4%, 8.3%
Clinical vulnerability								
Not clinically vulnerable	57 (34%)	27%, 42%	3110 (58%)	57%, 59%	21 (24%)	16%, 35%	1488 (51%)	50%, 53%
Clinically vulnerable	52 (31%)	24%, 39%	1583 (30%)	28%, 31%	23 (27%)	18%, 38%	961 (33%)	31%, 35%
Clinically extremely vulnerable	57 (34%)	27%, 42%	671 (13%)	12%, 13%	42 (49%)	38%, 60%	449 (15%)	14%, 17%
Solid organ cancer (non-Haem)	19 (11%)	7.2%, 18%	444 (8.3%)	7.6%, 9.1%	13 (15%)	8.6%, 25%	290 (10%)	9.0%, 11%
Haematological cancer	15 (9.0%)	5.3%, 15%	38 (0.7%)	0.51%, 1.0%	7 (8.1%)	3.6%, 17%	24 (0.8%)	0.54%, 1.2%
Haematological non-malignant	4 (2.4%)	0.77%, 6.4%	36 (0.7%)	0.48%, 0.94%	4 (4.7%)	1.5%, 12%	17 (0.6%)	0.35%, 1.0%
Asthma	26 (16%)	11%, 22%	905 (17%)	16%, 18%	7 (8.1%)	3.6%, 17%	495 (17%)	16%, 19%
COPD	11 (6.6%)	3.5%, 12%	160 (3.0%)	2.6%, 3.5%	8 (9.3%)	4.4%, 18%	114 (3.9%)	3.3%, 4.7%
Severe respiratory disease	7 (4.2%)	1.9%, 8.8%	290 (5.4%)	4.8%, 6.1%	4 (4.7%)	1.5%, 12%	145 (5.0%)	4.3%, 5.9%
Ischaemic heart disease	13 (7.8%)	4.4%, 13%	238 (4.4%)	3.9%, 5.0%	10 (12%)	6.0%, 21%	185 (6.4%)	5.5%, 7.4%
Hypertension	65 (39%)	32%, 47%	1406 (26%)	25%, 27%	28 (33%)	23%, 44%	956 (33%)	31%, 35%
Heart failure	5 (3.0%)	1.1%, 7.3%	21 (0.4%)	0.25%, 0.61%	4 (4.7%)	1.5%, 12%	20 (0.7%)	0.43%, 1.1%
Chronic viral infection	1 (0.6%)	0.03%, 3.8%	29 (0.5%)	0.37%, 0.79%	0 (0%)	0.00%, 5.3%	9 (0.3%)	0.15%, 0.61%
Type 1 DM	2 (1.2%)	0.21%, 4.7%	27 (0.5%)	0.34%, 0.74%	2 (2.3%)	0.40%, 8.9%	15 (0.5%)	0.30%, 0.87%
Type 2 DM	10 (6.0%)	3.1%, 11%	237 (4.4%)	3.9%, 5.0%	12 (14%)	7.7%, 23%	170 (5.9%)	5.1%, 6.8%
Stroke	7 (4.2%)	1.9%, 8.8%	90 (1.7%)	1.4%, 2.1%	0 (0%)	0.00%, 5.3%	66 (2.3%)	1.8%, 2.9%
Neurological condition	10 (6.0%)	3.1%, 11%	106 (2.0%)	1.6%, 2.4%	4 (4.7%)	1.5%, 12%	72 (2.5%)	2.0%, 3.1%
Mental ill-health	2 (1.2%)	0.21%, 4.7%	82 (1.5%)	1.2%, 1.9%	4 (4.7%)	1.5%, 12%	39 (1.3%)	1.0%, 1.9%
Liver condition	5 (3.0%)	1.1%, 7.3%	98 (1.8%)	1.5%, 2.2%	3 (3.5%)	0.91%, 11%	55 (1.9%)	1.4%, 2.5%
Inflammatory conditions	24 (14%)	9.7%, 21%	325 (6.1%)	5.4%, 6.7%	14 (16%)	9.5%, 26%	232 (8.0%)	7.1%, 9.1%
Chronic kidney disease	10 (6.0%)	3.1%, 11%	55 (1.0%)	0.78%, 1.3%	4 (4.7%)	1.5%, 12%	54 (1.9%)	1.4%, 2.4%
Immunosuppressive Drugs								
Steroids (long course)	16 (9.6%)	5.8%, 15%	72 (1.3%)	1.1%, 1.7%	7 (8.1%)	3.6%, 17%	36 (1.2%)	0.88%, 1.7%
DMARD	23 (14%)	9.2%, 20%	85 (1.6%)	1.3%, 2.0%	15 (17%)	10%, 27%	69 (2.4%)	1.9%, 3.0%
MAB	13 (7.8%)	4.4%, 13%	25 (0.5%)	0.31%, 0.70%	5 (5.8%)	2.2%, 14%	27 (0.9%)	0.63%, 1.4%

^a^Confidence Intervals for the percentage.

Notably, less than 10 individuals remained seronegative after Dose 2, with >95% of those who were seronegative at ≥28 days after Dose 1 becoming seropositive at ≥28 days after Dose 2 (data not presented in the tables due to small numbers).

### S-antibody levels by clinical risk factor

8356 individuals, including 5568 (67%) ChAdOx1 recipients and 2788 (33%) BNT162b2 recipients were included in the multivariable analyses of S-antibody levels at ≥28 days after Dose 2, by different clinical risk factors (Table 3). Of these 4821 (58%) were female, 4031 (48%) were ≥65 years old, and 7720 (92%) were of White British ethnicity. The median time since Dose 2 was 81 (62, 99) days, and the median Dose 1–2 interval was 77 (71, 78) days.

**Table 3:** Beta coefficients, 95% confidence intervals, and associated unadjusted *p* values (from two-tailed *t*-tests) derived from a single multivariable linear regression model of demographic and vaccination factors associated with log(n) Spike-antibody levels at ≥28 days after Dose 2. These covariates were subsequently included in the adjusted linear regression models examining the association between each clinical risk factor and log(n) Spike-antibody levels at ≥28 days after receiving Dose 2

	n/N (%)	Adjusted linear regression		
	*N* = 8356	Beta	95% CI^a^	*p* value
Median log(n) S-antibody (IQR)	6.95 (6.11, 7.85)			
Age group (years)				
45–64 (ref)	3572 (43%)	–	–	
18–24	61 (0.7%)	0.32	0.06, 0.59	0.017
25–44	692 (8.3%)	0.10	0.01, 0.18	0.034
65+	4031 (48%)	−0.05	−0.10, 0.00	0.075
Sex				
Male	3535 (42%)	–	–	
Female	4821 (58%)	0.08	0.04, 0.13	<0.001
Dose 2 vaccine type				
ChAdOx1-S	5568 (67%)	–	–	
BNT162b2	2788 (33%)	1.7	1.7, 1.8	<0.001
Dose 1–2 interval (days)	77 (71, 78)	0.01	0.01, 0.01	<0.001
Time since Dose 2 (days)	81 (62, 99)	−0.01	−0.01, −0.01	<0.001
Ethnicity				
White British	7720 (92%)	–	–	
Minority ethnic	636 (7.6%)	0.02	−0.06, 0.11	0.6

^a^CI = Confidence Interval.

All analyses adjusted for the following covariates: days since Dose 2, which appeared to be inversely associated with log(n) S-antibody levels (−0.01 [−0.01, −0.01]; *p* < 0.001); dose interval in days, which was positively associated (0.01 [0.01, 0.01]; *p* < 0.001); minority ethnicity, which was not associated (0.02 [−0.06, 0.11]; *p* = 0.6); for female sex, which was positively associated (0.08 [0.04, 0.13]; *p* < 0.001); age, where the youngest two age group (18–24 years: 0.32 [0.06, 0.59], *p* = 0.017; 25–44 years: 0.10 [0.01, 0.18], *p* = 0.034) had higher log(n) S-antibody levels than the reference group of 45–64 years, with no significant difference seen for the oldest group though the estimates were suggestive of an age-based gradient; and for BNT162b2 vaccine type, which was strongly positively associated compared to ChAdOx1-S (1.7 [1.7, 1.8]; *p* < 0.001) (Table 3).

**Fig. 2:**
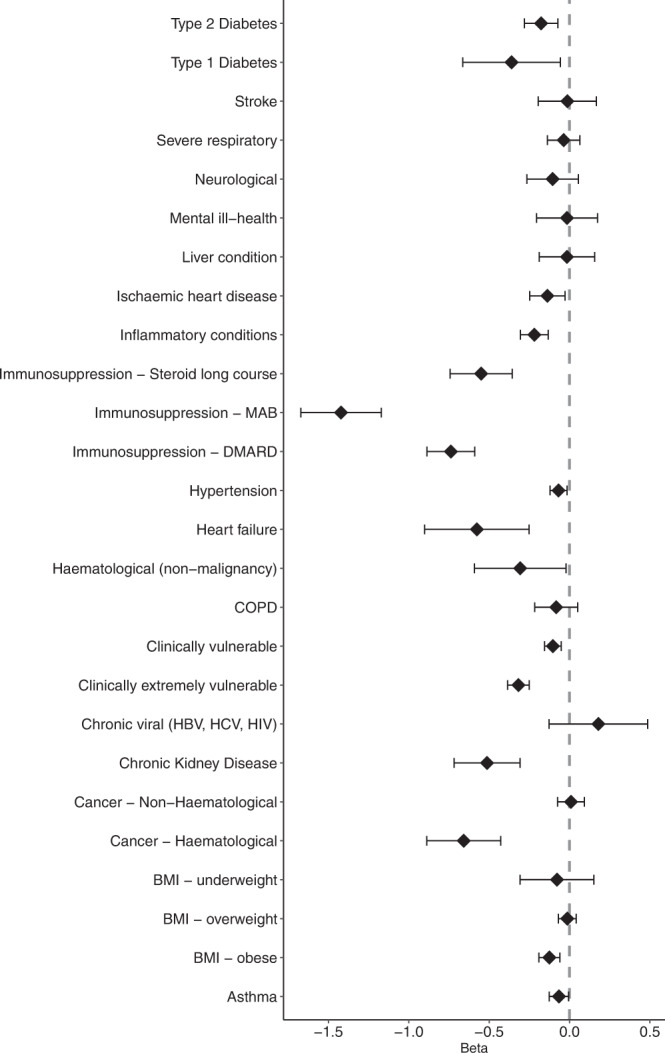
Dose 2 S-antibody levels linear regression forest plot. Beta coefficients and 95% confidence intervals, which are displayed as point estimates and associated error bars, derived from adjusted linear regression models for the effect of each clinical risk factor (investigated using a separate model) on log(n) S-antibody levels (U/ml) at ≥28 days after Dose 2. Each model controls for age, sex, ethnicity, vaccine type, dose interval and time since vaccination. Numbers included in each clinical group are as follows: Type 2 Diabetes *n* = 411, Type 1 Diabetes *n* = 46, Stroke *n* = 131, Severe respiratory *n* = 436, Neurological *n* = 168, Mental ill-health *n* = 119, Liver condition *n* = 144, Ischaemic heart disease *n* = 374, Inflammatory conditions *n* = 608, Immunosuppression Steroid long course *n* = 114, Immunosuppression MAB *n* = 67, Immunosuppression DMARD *n* = 194, Hypertension *n* = 2208, Heart failure *n* = 40, Haematological (non-malignancy) *n* = 52, COPD = 244, Clinically vulnerable *n* = 2319, Clinically extremely vulnerable *n* = 1144, Chronic viral (HBV, HCV, HIV) *n* = 45, Chronic kidney disease *n* = 101, Cancer Non-Haematological *n* = 666, Cancer Haematological *n* = 80, BMI underweight n = 82, BMI overweight *n* = 2619, BMI obese *n* = 1464, Asthma *n* = 1375.

Results from the main multivariable models demonstrated that several of the examined clinical risk factors were inversely associated with antibody levels, and none were positively associated (Fig. 2, Supplementary Table S3). MAB therapies (−1.42 [−1.67, −1.17]), DMARDs (−0.72 [−0.87, −0.58]), haematological cancer (−0.66 [−0.89, −0.43]), and long courses of steroids (−0.55 [−0.74, −0.36]), were most strongly inversely associated with log(n) S-antibody levels.

A number of more common conditions also appeared to be inversely associated with S-antibody response in the main analysis (Fig. 2, Supplementary Table S3), which were confirmed through the sensitivity analysis (Supplementary Fig. S2, Table S4): ischaemic heart disease (−0.15 [−0.26, −0.04]), obesity when compared to normal BMI (−0.12 [−0.19, −0.06]), Type 1 Diabetes (−0.32 [−0.62, −0.02]), Type 2 Diabetes (−0.15 [−0.25, −0.05]), heart failure (−0.55 [−0.87, −0.24]), CKD (−0.43 [−0.64, −0.23]), hypertension (−0.06 [−0.11, −0.01]). While inflammatory conditions were initially inversely associated with S-antibody levels (−0.22 [−0.31, −0.13]) in the main model, this association was no longer seen in the sensitivity analysis (−0.02 [−0.11, 0.07]).

Notably, JCVI definitions of clinical vulnerability to COVID-19 were strongly inversely associated with S-antibody levels in both models, with a much stronger association seen for the Clinically Extremely Vulnerable group (−0.32 [−0.38, −0.25]) than the Clinically Vulnerable group (−0.11 [−0.16, −0.05]) in the main model, however estimates became more similar in the sensitivity analysis (−0.15 [−0.22, −0.07] vs −0.10 [−0.15, −0.05]), after adjustment for immunosuppressive therapies and conditions, which form part of the criteria for extreme clinical vulnerability.

## Discussion

Findings from this large community cohort without evidence of prior infection, show that Spike-antibody levels rose earlier following the first dose of a Pfizer vaccine compared with an AstraZeneca vaccine, however S-antibody levels were comparable from 8 weeks onwards. The vast majority of individuals seroconverted to Spike from 4 weeks after one dose of either vaccine and S-antibody levels remained relatively stable for up to 8 weeks. S-antibody levels achieved their peak at around 2 weeks following the second dose, with the peak for BNT162b2 being significantly higher than that for ChAdOx1-S recipients, though antibody levels started declining in a similar fashion for both vaccine types from 4 weeks onwards, until at least 16 weeks after the second dose. While almost all individuals who did not seroconvert after the first dose did become seropositive after the second dose, significant disparities in S-antibody levels were nonetheless evident between different demographic and clinical groups. Female sex, younger age, longer dose interval, and BNT162b2 were positively associated with S-antibody response, while longer time since vaccination was inversely associated. As expected, diagnoses or therapies affecting the immune system, such as haematological malignancies, long steroid courses, and immune-modulating drugs were strongly inversely associated with S-antibody levels. Importantly, we show that S-antibody levels were also inversely associated with common conditions including diabetes, obesity, hypertension and cardiac conditions, and chronic kidney disease. Of note, the UK JCVI’s clinical vulnerability categorisations, which have previously been used for vaccine prioritisation and shielding guidelines, were themselves strongly predictive of lower antibody responses to vaccination.

A study of a large healthcare worker cohort found that seroconversion rates were 99.5% (2706/2720) and 97.1% (864/890) at >14 days after a single dose of BNT162b2 and ChAdOx1, respectively^27^. The slightly lower seroconversion rates observed in our cohort could be attributed to the older average age and higher prevalence of comorbidities, and thus a reduced healthy-worker bias. Conversely, seroconversion rates appear substantially higher in our cohort than those reported by the REACT2 study^28^, which found 84.1% (82.2, 85.9) seroconversion ≥21 days after a dose of BNT162b2; though this may be attributable to differences in assay sensitivities. A large observational study including 38,262 SARS-CoV-2-naive individuals (from a community cohort enrolled in the UK’s national COVID-19 Infection Survey), modelled the probability of seroconversion to Spike following a single dose vaccination^29^. Similar to our findings, they reported slower rise in S-antibody titres, following ChAdOx1 compared with BNT162b2; also in line with our findings, differences between the two vaccines attenuated at later time points following the first dose.

Trial and observational data indicate greater immunogenicity in younger compared to older adults^29–31^, in females compared to males^29,32^, and with longer dose intervals^11,12^, which are also reflected in our findings. As expected, those groups with immunosuppression due to disease or therapy had markedly lower S-antibody levels after Dose 2. Our results are concordant with findings from studies focusing on patients with haematological malignancies^19,20^ and those with immunosuppression^13–18^. However there is as yet little data relating to more common conditions, and our results relating to those with cardio-metabolic diseases, as well as groups classified as clinically vulnerable, constitute some of the earliest and most robust evidence of lower vaccine immunogenicity from large cohorts living with these conditions. These findings highlight the need to further investigate underlying mechanisms of poor or dysregulated immune responses, and any consequent disparities in vaccine efficacy, amongst adults with high cardio-metabolic risk, who have also suffered high levels of morbidity and mortality due to COVID-19.

Our findings provide further immunological insights to support real-world data on vaccine effectiveness. Reports of single-dose vaccine efficacy from the UK^8,9^ and Israel^33^ indicate onset of protection from 2–4 weeks after a single dose, with slightly longer taken to reach peak efficacy with ChAdOx1 than BNT162b2. These findings mirror the Dose 1 immunogenicity data reported here. Following two doses, emerging data suggest lower Spike- and neutralising-antibody titres are associated with more breakthrough infections^34,35^. Furthermore, there is accumulating data suggesting that vaccine efficacy against infection varies between age groups^36,37^ and by clinical vulnerability^38^, though data is more mixed regarding dose interval^36,39^. Regarding vaccine type, earlier evidence from the UK, where both BNT162b2 and ChAdOx1-S have been widely used, indicated similar efficacy^9,40^. This may be because the S-antibodies measured by the Roche assay used in our study are not a perfect correlate of neutralising antibodies, or that other aspects of the immune response, such as cellular and innate components, are better stimulated by the ChAdOx1 vaccine platform, contributing to its high protective efficacy. Our findings are in line with the accumulating evidence for waning of immunity over time, both in terms of antibody levels^29,41^, and in terms of protective efficacy of vaccination^7,36,37^ that is seen across all groups to different extents. The consistency of waning across both vaccine effectiveness estimates and immunological data suggests that the observed reductions in efficacy are unlikely to be wholly due to the impact of immunity-evading variants and are likely to be at least partly explained by reductions in protective antibodies over time. Interestingly vaccine efficacy declines more markedly for ChAdOx1-S recipients, as well in the older and more clinically vulnerable, both over time and against the Delta variant^36,37,42,43^, which could be related to the lower peak antibody titres we have observed in these groups. It remains to be seen whether antibody levels plateau over longer periods of follow-up, and whether similar waning is also seen after booster doses. It seems likely, however, that the most clinically vulnerable groups who were prioritised for initial vaccination and subsequently for boosting, particularly those who received ChAdOx1-S, could have the lowest antibody levels in future months, as a result of attenuated antibody responses at baseline, compounded by antibody waning over time since vaccination.

The strengths of this study include the large community cohort, which spans many demographic groups, and includes large numbers of individuals with common chronic conditions as well as capturing a substantial number of those with rarer diagnoses. We employed a highly sensitive, widely used, validated commercial assay allowing these data to be easily understood, replicated, and informative for clinical practitioners. We were able to use reliable routinely collected data on vaccination, supplemented with self-reported data. Finally, we conducted a sensitivity analysis that adjusted for the most strongly associated immunosuppressive conditions and therapies, in order to robustly assess the associations between wider range of clinical conditions and vaccine responses. Limitations include the use of serological data from a large number of individuals at different time points to infer overall trends in antibody levels over time; the possible misclassification of previously infected individuals who did not seroconvert to Nucleocapsid, those who sero-reverted, or those with early infection; and the collection of clinical data at two time points only, which may have led to misclassification of diagnoses or therapies that affected vaccine responses but were missed by the questionnaires due to recent changes. Importantly, there is likely to be a substantial degree of overlap between clinical conditions, for example between cardio-metabolic conditions diabetes, obesity, hypertension; between ischaemic heart disease and heart failure; and also between these conditions and CKD and stroke; therefore the findings should not be taken as indicative of causation, but should be interpreted as population-level information on clinical risk, intended to guide individual/clinical group risk stratification. Finally, it is important to acknowledge that booster vaccination programmes are now well underway in the U.K. and many other countries, therefore further data is needed to ascertain whether these clinical disparities in vaccine responses persist beyond the primary course, and the impact of heterologous booster regimens.

In conclusion, our data indicate very high rates of seroconversion to Spike and high S-antibody levels following a primary course of either vaccine, in individuals with no evidence of prior infection. Despite higher peak responses following BNT162b2 compared to ChAdOx1-S, both vaccines demonstrate similar, sustained rates of antibody waning from 8 weeks after a second dose. Furthermore, despite near-total seroconversion, significant disparities in S-antibody levels are still seen after Dose 2 for several clinical risk groups, including those with common conditions such as diabetes, obesity, hypertension, heart disease, and chronic kidney disease. Given the accumulating evidence of waning antibody levels and vaccine effectiveness over time, and the emergence of immunity-evading variants Delta and Omicron, it is likely that clinically vulnerable groups, who have been vaccinated earliest and have the lowest antibody responses, will be at highest risk of COVID-19 morbidity and mortality going forwards. As updated versions of COVID-19 vaccines specific to new variants reach licensure, it seems advisable to continue to prioritise the clinically vulnerable groups, who will remain most at risk.

## Methods

The Virus Watch study complies with all relevant ethical regulations and the study protocol has been approved by the Hampstead NHS Health Research Authority Ethics Committee. Ethics approval number —20/HRA/2320. Informed consent was obtained from participants.

### Study design and setting

This analysis was conducted as part of Virus Watch––a household community cohort study of acute respiratory infections in England & Wales that started recruitment in June 2020^26^. We used a range of recruitment methods: generation of a random list of residential address lists using the Royal Mail Post Office Address File, that were sent recruitment postcards; placement of social media adverts on Facebook and Twitter; sending of SMS messages and letters to potential participants via their General Practitioners. Participants were followed-up weekly by email with a link to a survey which asked about symptoms that could indicate COVID-19 disease, SARS-CoV-2 test results received from outside the study (e.g. via the Second Generation Surveillance System system), and details of vaccinations received against COVID-19. A further round of recruitment was undertaken in October 2020–January 2021 to enrich the cohort with minority ethnic groups. Invitations to participate in monthly antibody testing were sent to enrolled households starting from February 2021, starting with those already vaccinated and those in clinical priority groups, so as to commence serum sampling as close to the time of vaccination as possible. Consenting adults (18+ years) provided monthly capillary blood samples between 24 February 2021 and 17 December 2021. Demographic and clinical data were collected at enrolment and through a further detailed clinical survey in May 2021.

Capillary blood samples (400–600 microlitres) were self-collected by participants using an at-home collection kit manufactured by Thriva Ltd [https://thriva.co/]. Completed kits were returned by participants using pre-paid envelopes and priority postage boxes to UKAS-accredited laboratories. The sampling date was taken as being two days prior to the date of receipt by the laboratory. Serological testing was undertaken using Roche’s Elecsys Anti-SARS-CoV-2 assays targeting total immunoglobulin (Ig) to the Nucleocapsid (N) protein (positive defined as ≥1.0 cut-off index [COI]) or to the receptor binding domain in the S1 subunit of the Spike protein (S) (range 0.4–250 units per millilitre [U/ml] without serum dilution; 0.4–25,000 U/ml with 1:100 serum dilution; seropositive defined as ≥0.8 U/ml) (Roche Diagnostics, Basel, Switzerland). At the manufacturer-recommended cut-off of ≥1.0 COI the Nucleocapsid assay is highly sensitive and specific for antibodies produced by wild-type SARS-CoV2 infection^44,45^.

Data on vaccination dates, dose number, and type were obtained through pseudonymised linkage via NHS Digital with routinely collected national vaccination records within the National Immunisation Monitoring System (NIMS). Additionally, where linked data was not available, self-reported vaccination status was collected through the weekly Virus Watch questionnaire (retrospectively and prospectively from 11 January 2021).

### Participants

Within the Virus Watch full cohort (Supplementary Table S1), eligible individuals were defined as those aged ≥18 years, with a valid England or Wales postcode, a complete postal address registered at enrolment, complete sex and ethnicity information, and not already enrolled in longitudinal point-of-care antibody testing as part of index case investigations^26^. Eligible individuals were provided with a participant information sheet and gave valid, informed consent through an electronic REDCap form. Recipients of vaccines other than BNT162b2 and ChAdOx1-S were not included due to very small numbers.

Descriptive analyses included samples taken from the date of first vaccination onwards (Dose 1 samples) or from the date of second vaccination onwards (Dose 2 samples), with individuals able to contribute more than one sample; samples are categorised by days since Dose 1 or Dose 2. The multivariable analysis included only the first sample for each individual taken at ≥28 days after Dose 2, and prior to any third booster dose. Evidence of natural infection was defined as seropositivity against the Nucleocapsid protein; samples meeting this criterion were excluded from all analyses so as to investigate vaccine-induced antibody responses only, without the added effects of infection. If an individual became N-seropositive partway through the follow-up period, their N-seronegative samples would still be included in the relevant analyses, with any later N-seropositive samples excluded. Individuals with missing information on vaccination date or type, on both routine and self-reported data, were excluded from the analysis. Samples with void results from either assay were excluded from the analyses. Individuals with invalid dose interval or dose interval less than 21 days were excluded from the Dose 2 analyses.

At the start of the study, serum samples were tested without dilution as antibody levels following Dose 1 remained well below the 250 U/ml upper limit of the assay. Following roll-out of Dose 2, the testing protocol was changed to incorporate 1:100 serum dilution in order to expand the upper limit of the assay to 25,000 U/ml, as S-antibody levels following complete vaccination were substantially higher than after partial vaccination. Dose 2 samples tested without 1:100 dilution were therefore excluded from all analyses. We were not able to include data from samples taken following a third ‘booster’ dose due to the S-antibody levels in these samples routinely exceeding the new 25,000 U/ml upper limit of the assay.

### Exposures

Binary exposure variables were based on the following clinical risk factors: active diagnoses of Type 1 Diabetes, Type 2 Diabetes, Stroke, Neurological conditions (excluding stroke), Mental ill-health, Liver conditions, Inflammatory conditions, Hypertension, Heart Failure, Ischaemic Heart Disease, Asthma, Chronic Obstructive Pulmonary Disease (COPD), Severe Respiratory Disease, Chronic Kidney Disease (CKD), Haematological non-malignant conditions, Chronic viral infections (Hepatitis B and C and HIV), Underweight, Overweight, Obesity, Solid Organ Cancer (non-haematological), and Haematological Cancer; the following therapies: Steroids (long course), Disease-Modifying Anti-Rheumatic Drugs (DMARD), and Monoclonal Antibodies (MAB) at any time during the study; and finally the UK JCVI definitions used for vaccine prioritisation^4^: Clinically Vulnerable and Clinically Extremely Vulnerable. We calculated BMI using self-reported height and weight data. Please see Supplementary Table S2 for further information on how the grouped categories were defined.

### Outcomes

The main outcome variable was defined as the natural logarithm (log[n]) of the Spike antibody level (U/ml).

### Covariates

Age was grouped into 18–24, 25–44, 45–64, and ≥65 years categories. Ethnicity data were collapsed into a binary variable of White British and Minority Ethnic. Sex was limited to a Male and Female binary variable, with other categories excluded due to small numbers. Vaccine type was a binary variable of BNT162b2 (Pfizer) or ChAdOx1-S (Oxford-AstraZeneca). Time since Dose 2 vaccination and Dose 1 to Dose 2 interval were coded continuously and represented the number of days for each variable.

### Statistical analysis

In the descriptive analyses, we plotted the geometric mean of S-antibody levels, including samples below the cut-off for binary seropositivity, for each week after either the first or second dose of vaccination, along with the 95% confidence intervals around the means. We also calculated the percentages of individuals who remained seronegative and those who became seropositive at ≥28 days after Dose 1, along with the 95% confidence intervals around the serostatus percentage estimates.

In the multivariable models, we used linear regression to compare log(n) S-antibody levels between those with and without each clinical risk factor, controlling for age, sex, ethnicity, dose interval and time since vaccination, deriving the Beta coefficient (based on log(n) antibody levels) and associated 95% confidence intervals for each exposure. We also conducted a sensitivity analysis that additionally adjusted for key immunosuppressive conditions and therapies (haematological cancers, long courses of steroids, DMARDs, and MABs) that were found to be strongly negatively associated with Spike-antibody levels in the main analysis, in order to account for any confounding effects and therefore better study the other clinical conditions of interest.

Data were collected using REDCap 12.4.0 and analyses were conducted in R 4.0.3. The study is registered with ISRCTN: 10.1186/ISRCTN32077121.

### Reporting summary

Further information on research design is available in the Nature Research Reporting Summary linked to this article.

## Supplementary information

Supplementary Information

Reporting Summary

## Data Availability

The raw data used in this study have been deposited in the ONS Secure Research Service. The data are available under restricted access as they contain sensitive health data. Access can be obtained by ONS Secure Research Service.

## Code availability

Code for these analyses are available from the corresponding author upon request.

## References

[1] World Health Organization. Coronavirus (COVID-19) Dashboard. https://covid19.who.int/ (2021). Accessed 3 March 2022.

[2] Independent report - Optimising the COVID-19 vaccination programme for maximum short-term impact. 326 January 2021. https://www.gov.uk/government/publications/prioritising-the-first-covid-19-vaccine-dose-jcvi-statement/optimising-the-covid-19-vaccination-programme-for-maximum-short-term-impact. Accessed 3 March 2022.

[3] Oliveira SC, de Magalhães MTQ, Homan EJ (2020). Immunoinformatic analysis of SARS-CoV-2 nucleocapsid protein and identification of COVID-19 vaccine targets. Front. Immunol..

[4] Independent report - Priority groups for coronavirus (COVID-19) vaccination: advice from the JCVI. 30 December 2020. https://www.gov.uk/government/publications/priority-groups-for-coronavirus-covid-19-vaccination-advice-from-the-jcvi-30-december-2020. Accessed 3 March 2022.

[5] UK Health Security Agency. Coronavirus (COVID-19) in the UK dashboard. https://coronavirus.data.gov.uk/. Accessed 3 March 2022.

[6] Polack FP (2020). Safety and efficacy of the BNT162b2 mRNA Covid-19 vaccine. N. Engl. J. Med..

[7] Katikireddi SV (2022). Two-dose ChAdOx1 nCoV-19 vaccine protection against COVID-19 hospital admissions and deaths over time: a retrospective, population-based cohort study in Scotland and Brazil. Lancet.

[8] Lopez Bernal, J. et al. Effectiveness of the Pfizer-BioNTech and Oxford-AstraZeneca vaccines on covid-19 related symptoms, hospital admissions, and mortality in older adults in England: test negative case-control study. *Bmj*. n1088, 10.1136/bmj.n1088 (2021). 10.1136/bmj.n1088 PMC8116636 33985964

[9] Pritchard E (2021). Impact of vaccination on new SARS-CoV-2 infections in the United Kingdom. Nat. Med..

[10] Vasileiou E (2021). Interim findings from first-dose mass COVID-19 vaccination roll-out and COVID-19 hospital admissions in Scotland: a national prospective cohort study. Lancet (Lond., Engl.).

[11] Voysey M (2021). Single-dose administration and the influence of the timing of the booster dose on immunogenicity and efficacy of ChAdOx1 nCoV-19 (AZD1222) vaccine: a pooled analysis of four randomised trials. Lancet.

[12] Grunau B (2022). Immunogenicity of extended mRNA SARS-CoV-2 vaccine dosing intervals. JAMA - J. Am. Med. Assoc..

[13] Boyarsky BJ (2021). Antibody response to 2-dose sars-cov-2 mrna vaccine series in solid organ transplant recipients. JAMA - J. Am. Med. Assoc..

[14] Rabinowich, L. et al. Low immunogenicity to SARS-CoV-2 vaccination among liver transplant recipients. *J. Hepatol*. 10.1016/j.jhep.2021.04.020 (2021). 10.1016/j.jhep.2021.04.020 PMC8058047 33892006

[15] Marinaki, S. et al. Immunogenicity of SARS‐CoV‐2 BNT162b2 vaccine in solid organ transplant recipients. *Am. J. Transplant*. ajt.16607 10.1111/ajt.16607 (2021). 10.1111/ajt.16607 PMC8250574 33864722

[16] Deepak P (2021). Effect of Immunosuppression on the Immunogenicity of mRNA Vaccines to SARS-CoV-2. Ann. Intern. Med..

[17] Kennedy, N. A. et al. Infliximab is associated with attenuated immunogenicity to BNT162b2 and ChAdOx1 nCoV-19 SARS-CoV-2 vaccines in patients with IBD. *Gut* gutjnl-2021-324789 10.1136/gutjnl-2021-324789 (2021). 10.1136/gutjnl-2021-324789 33903149

[18] Mahil SK (2022). Humoral and cellular immunogenicity to a second dose of COVID-19 vaccine BNT162b2 in people receiving methotrexate or targeted immunosuppression: a longitudinal cohort study. Lancet Rheumatol..

[19] Monin, L. et al. Safety and immunogenicity of one versus two doses of the COVID-19 vaccine BNT162b2 for patients with cancer: interim analysis of a prospective observational study. *Lancet. Oncol*. 1–14, 10.1016/S1470-2045(21)00213-8 (2021). 10.1016/S1470-2045(21)00213-8 PMC8078907 33930323

[20] Herishanu, Y. et al. Efficacy of the BNT162b2 mRNA COVID-19 vaccine in patients with chronic lymphocytic leukemia. *Blood*10.1182/blood.2021011568 (2021). 10.1182/blood.2021011568 PMC8061088 33861303

[21] Docherty, A. B. et al. Features of 20 133 UK patients in hospital with covid-19 using the ISARIC WHO Clinical Characterisation Protocol: prospective observational cohort study. *BMJ* m1985 10.1136/bmj.m1985 (2020). 10.1136/bmj.m1985 PMC7243036 32444460

[22] Yates, T. et al. Obesity, ethnicity and risk of critical care, mechanical ventilation and mortality in patients admitted to hospital with COVID‐19: Analysis of the ISARIC CCP‐UK cohort. *Obesity* oby.23178 10.1002/oby.23178 (2021). 10.1002/oby.23178 PMC8251439 33755331

[23] McGurnaghan SJ (2021). Risks of and risk factors for COVID-19 disease in people with diabetes: a cohort study of the total population of Scotland. Lancet Diabetes Endocrinol..

[24] Mathur R (2021). Ethnic differences in SARS-CoV-2 infection and COVID-19-related hospitalisation, intensive care unit admission, and death in 17 million adults in England: an observational cohort study using the OpenSAFELY platform. Lancet.

[25] Arnold, J., Winthrop, K. & Emery, P. COVID-19 vaccination and antirheumatic therapy. *Rheumatology* 1–7 10.1093/rheumatology/keab223 (2021). 10.1093/rheumatology/keab223 PMC7989162 33710296

[26] Hayward A (2021). Risk factors, symptom reporting, healthcare-seeking behaviour and adherence to public health guidance: protocol for Virus Watch, a prospective community cohort study. BMJ Open.

[27] Eyre DW (2021). Quantitative SARS-CoV-2 anti-spike responses to Pfizer–BioNTech and Oxford–AstraZeneca vaccines by previous infection status. Clin. Microbiol. Infect..

[28] Ward, H. *et al*. REACT-2 Round 5: increasing prevalence of SARS-CoV-2 antibodies demonstrate impact of the second wave and of vaccine roll-out in England. *medRxiv* 2021.02.26.21252512 (2021).

[29] Wei J (2021). Antibody responses to SARS-CoV-2 vaccines in 45,965 adults from the general population of the United Kingdom. Nat. Microbiol..

[30] Walsh EE (2020). Safety and Immunogenicity of Two RNA-Based Covid-19 Vaccine Candidates. N. Engl. J. Med..

[31] Tut G (2021). Profile of humoral and cellular immune responses to single doses of BNT162b2 or ChAdOx1 nCoV-19 vaccines in residents and staff within residential care homes (VIVALDI): an observational study. Lancet Heal. Longev..

[32] Demonbreun AR (2021). COVID-19 mRNA Vaccination Generates Greater Immunoglobulin G Levels in Women Compared to Men. J. Infect. Dis..

[33] Dagan N (2021). BNT162b2 mRNA Covid-19 Vaccine in a Nationwide Mass Vaccination Setting. N. Engl. J. Med..

[34] Aldridge, R. W. et al. Waning of SARS-CoV-2 antibodies targeting the Spike protein in individuals post second dose of ChAdOx1 and BNT162b2 COVID-19 vaccines and risk of breakthrough infections: analysis of the Virus Watch community cohort. *medRxiv* 2021.11.05.21265968 (2021).

[35] Khoury, D. S. et al. Neutralizing antibody levels are highly predictive of immune protection from symptomatic SARS-CoV-2 infection. *Nat. Med*. 10.1038/s41591-021-01377-8 (2021). 10.1038/s41591-021-01377-8 34002089

[36] Pouwels KB (2021). Effect of Delta variant on viral burden and vaccine effectiveness against new SARS-CoV-2 infections in the UK. Nat. Med..

[37] Andrews N (2022). Duration of protection against mild and severe disease by Covid-19 vaccines. N. Engl. J. Med..

[38] McKeigue, P. M. et al. Efficacy of two doses of COVID-19 vaccine against severe COVID-19 in those with risk conditions and residual risk to the clinically extremely vulnerable: the REACT-SCOT case-control study. *medRxiv* 2021.09.13.21262360 (2021).

[39] Amirthalingam G (2021). Serological responses and vaccine effectiveness for extended COVID-19 vaccine schedules in England. Nat. Commun..

[40] Hulme, W. J. et al. Comparative effectiveness of ChAdOx1 versus BNT162b2 COVID-19 vaccines in Health and Social Care workers in England: a cohort study using OpenSAFELY. *medRxiv* 2021.10.13.21264937 (2021). 10.1136/bmj-2021-068946 PMC9295078 35858680

[41] Levin EG (2021). Waning immune humoral response to BNT162b2 Covid-19 vaccine over 6 months. N. Engl. J. Med..

[42] Sheikh A, McMenamin J, Taylor B, Robertson C (2021). SARS-CoV-2 Delta VOC in Scotland: demographics, risk of hospital admission, and vaccine effectiveness. Lancet.

[43] McKeigue, P. M. et al. Efficacy of vaccination against severe COVID-19 in relation to Delta variant and time since second dose: the REACT-SCOT case-control study. *medRxiv* 2021.09.12.21263448 (2021).

[44] Muench, P. et al. Development and Validation of the Elecsys Anti-SARS-CoV-2 Immunoassay as a Highly Specific Tool for Determining Past Exposure to SARS-CoV-2. *J. Clin. Microbiol*. **58**, (2020). 10.1128/JCM.01694-20 PMC7512151 32747400

[45] Ainsworth M (2020). Performance characteristics of five immunoassays for SARS-CoV-2: a head-to-head benchmark comparison. Lancet Infect. Dis..

