# Supplementary material for: Spike-antibody responses to COVID-19 vaccination by demographic and clinical factors in a prospective community cohort study
Source: Nat Commun. 2022 Oct 2;13:5780. doi: 10.1038/s41467-022-33550-z (PMC9526787; doi:10.1038/s41467-022-33550-z)
Supplement: Supplementary file 1 — Supplementary Information [file 41467_2022_33550_MOESM1_ESM.pdf]

Supplementary Materials

Virus Watch Collaborative – Consortium contributors' list, including current study authors.

Figure S1 – Study inclusion flow diagrams for **a)** Dose 1 analyses and **b)** Dose 2 analyses.

Table S1 – Characteristics of the full Virus Watch study cohort, the study sub-cohort who provided at least one serum sample with valid informed consent, and the sub-cohort with at least one valid serum sample available following Dose 2 (i.e. those included in the current study Dose 2 descriptive analysis).

Table S2 – Definitions of the grouped clinical risk factors used in multivariable linear regression analysis.

Table S3 – Main analysis beta coefficients, 95% confidence intervals, and associated unadjusted p-values (from two-tailed t-tests) for the association between each clinical risk factor and log(n) S-antibody levels (U/ml) from separate multivariable linear regression models adjusting for age, sex, ethnicity, vaccine type, dose interval, and time since vaccination (data underlying main paper Figure 2).

Figure S2 – Sensitivity analysis forest plot displaying the Beta coefficients and 95% confidence intervals, which are displayed as point estimates and associated error bars, derived from adjusted linear regression models for the effect of each clinical risk factor (investigated using a separate model) on log(n) S-antibody levels (U/ml) at ≥28 days after Dose 2. Each model controls for age, sex, ethnicity, vaccine type, dose interval, time since vaccination, and key immunosuppressive conditions and therapies shown to significantly affect vaccine response. Numbers included in each clinical group are as follows: Type 2 Diabetes n=411, Type 1 Diabetes n=46, Stroke n=131, Severe respiratory n=436, Neurological n=168, Mental ill-health n=119, Liver condition n=144, Ischaemic heart disease n=374, Inflammatory conditions n=608, Hypertension n=2208, Heart failure n=40, Haematological (non-malignancy) n=52, COPD=244, Clinically vulnerable n=2319, Clinically extremely vulnerable n=1144, Chronic viral (HBV, HCV, HIV) n=45, Chronic kidney disease n=101, Cancer Non-Haematological n=666, BMI underweight n=82, BMI overweight n=2619, BMI obese n=1464, Asthma n=1375.

Table S4 – Sensitivity analysis beta coefficients, 95% confidence intervals, and associated unadjusted p-values (from two-tailed t-tests) for the association between each clinical risk factor and log(n) S-antibody levels (U/ml) from separate multivariable linear regression models adjusting for age, sex, ethnicity, vaccine type, dose interval, time since vaccination, and key immunosuppressive conditions and therapies shown to significantly affect vaccine response (data underlying supplementary Figure S2).

Virus Watch Collaborative – consortium contributors' list

**Robert W Aldridge**	University College London, London, UK
**Anna Aryee**	University College London, London, UK
**Sarah Beale**	University College London, London, UK
**Isobel Braithwaite**	University College London, London, UK
**Thomas Byrne**	University College London, London, UK
**Tao Cheng**	University College London, London, UK
**Andrew Copas**	University College London, London, UK
**Ingemar Cox**	University College London, London, UK
**Wing Lam Erica Fong**	University College London, London, UK
**Ellen Fragaszy**	University College London, London, UK<br>London School of Hygiene & Tropical Medicine, London, UK
**Cyril Geismar**	University College London, London, UK
**Jo Gibbs**	University College London, London, UK
**Richard Gilson**	University College London, London, UK
**Pia Hardelid**	University College London, London, UK
**Andrew C Hayward**	University College London, London, UK
**Susan Hoskins**	University College London, London, UK
**Anne M Johnson**	University College London, London, UK
**Ben Killingley**	University of Nottingham, Nottingham, UK<br>University College London Hospital, London, UK
**Victoria Kjaergaard**	University College London, London, UK
**Jana Kovar**	University College London, London, UK
**Vasileios Lampos**	University College London, London, UK
**Yunzhe Liu**	University College London, London, UK
**Rachel A McKendry**	University College London, London, UK
**Susan Michie**	University College London, London, UK
**Faith Miller**	University College London, London, UK
**Eleni Nastouli**	University College London Hospital, London, UK<br>Francis Crick Institute, London, UK
**Annalan M D Navaratnam**	University College London, London, UK
**Aimee Serisier**	University College London, London, UK
**Chloe Siegele-Brown**	University College London, London, UK
**Madhumita Shrotri**	University College London, London, UK
**Colette Smith**	University College London, London, UK
**Moira Spyer**	Francis Crick Institute, London, UK
**Sam Tweed**	University College London, London, UK
**Alison Rodger**	Royal Free London NHS Foundation Trust, London, UK<br>University College London Hospital, London, UK
**Anna Sophie Weber**	University College London, London, UK
**Linda Wijlaars**	University College London, London, UK
**Alexei Yavlinsky**	University College London, London, UK

Figure S1a

	Included		Excluded	
Virus Watch full cohort	44,204	>	6,530	Incomplete demographic information
		>	23,395	No serum samples given
Serum sampling sub-cohort	14,279	>	1,620	Nucleocapsid seropositivity
	v			
	12,659	>	31	Spike result void
	v			
	12,628	>	38	Missing clinical information
	v			
	12,590	>	3,360	No Dose 1 sample
	v			
Included in Dose 1 descriptive analysis	9,230	>	716	No Dose 1 sample at 28+ days
	v			
Described in Dose 1 seronegative/seropositive table	8,514			

Figure S1b

	Included		Excluded	
Virus Watch full cohort	44,204	>	6,530	Incomplete demographic information
		>	23,395	No serum samples given
Serum sampling sub-cohort	14,279	>	1,620	Nucleocapsid seropositivity
	v			
	12,659	>	31	Spike result void
	v			
	12,628	>	38	Missing clinical information
	v			
	12,590	>	2,244	No Dose 2 sample
	v			
	10,346	>	1,801	No Dose 2 sample tested with 1:100 dilution
	v			
	8,545	>	74	Dose 1-2 interval invalid or <21 days
	v			
Included in Dose 2 descriptive analysis	8,471	>	115	No Dose 2 sample at 28+ days
	v			
Included in Dose 2 linear regression analyses	8,356			

**Table S1**	Virus Watch full cohort	Serum sampling sub-cohort	Dose 2 serum sample available
Characteristic	N = 44,204[1]	N = 14,279[1]	N = 8,471[1]
**Age group (years)**			
18-24	3,189 (7.2%)	308 (2.2%)	75 (0.9%)
25-44	10,540 (24%)	1,953 (14%)	770 (9.1%)
45-64	16,663 (38%)	5,782 (40%)	3,592 (42%)
65+	13,812 (31%)	6,236 (44%)	4,034 (48%)
Missing	0 (0%)	0 (0%)	
**Sex**			
Male	16,531 (37%)	6,147 (43%)	3,596 (42%)
Female	21,266 (48%)	8,108 (57%)	4,875 (58%)
Intersex	58 (0.1%)	15 (0.1%)	
Missing	6,349 (14%)	9 (<0.1%)	
**Ethnicity**			
White British	30,910 (70%)	12,817 (90%)	7,801 (92%)
Minority ethnic	6,661 (15%)	1,440 (10%)	670 (7.9%)
Prefer not to say	145 (0.3%)	22 (0.2%)	
Missing	6,488 (15%)	0 (0%)	
**BMI**			
Normal	11,577 (41%)	4,933 (41%)	3,024 (36%)
Obese	6,361 (23%)	2,576 (21%)	1,470 (17%)
Overweight	9,894 (35%)	4,349 (36%)	2,640 (31%)
Underweight	425 (1.5%)	143 (1.2%)	85 (1.0%)
Missing			1,252 (15%)
**Clinical vulnerability**			
Clinically extremely vulnerable	3,283 (7.4%)	1,866 (13%)	1,148 (14%)
Clinically vulnerable	10,478 (24%)	3,962 (28%)	2,324 (27%)
Not clinically vulnerable	30,443 (69%)	8,451 (59%)	4,999 (59%)
Solid organ cancer (non-Haem)	2,105 (4.8%)	1,021 (7.2%)	666 (7.9%)
Haematological cancer	263 (0.6%)	128 (0.9%)	80 (0.9%)
Haematological non-malignant condition	196 (0.4%)	90 (0.6%)	52 (0.6%)

**Table S1**	Virus Watch full cohort	Serum sampling sub-cohort	Dose 2 serum sample available
Characteristic	N = 44,204[1]	N = 14,279[1]	N = 8,471[1]
Asthma	5,727 (13%)	2,315 (16%)	1,384 (16%)
COPD	938 (2.1%)	410 (2.9%)	244 (2.9%)
Severe respiratory disease	1,143 (2.6%)	689 (4.8%)	437 (5.2%)
Ischaemic heart disease	1,364 (3.1%)	619 (4.3%)	374 (4.4%)
Hypertension	7,384 (17%)	3,498 (24%)	2,211 (26%)
Congestive heart failure	175 (0.4%)	77 (0.5%)	40 (0.5%)
Chronic viral (HBV, HCV, HIV)	188 (0.4%)	75 (0.5%)	45 (0.5%)
Type 1 DM	103 (0.2%)	64 (0.4%)	47 (0.6%)
Type 2 DM	1,193 (2.7%)	679 (4.8%)	411 (4.9%)
Stroke	508 (1.1%)	223 (1.6%)	131 (1.5%)
Neurological condition (excl. stroke)	712 (1.6%)	278 (1.9%)	168 (2.0%)
Mental ill-health	344 (0.8%)	199 (1.4%)	119 (1.4%)
Liver condition	559 (1.3%)	242 (1.7%)	145 (1.7%)
Inflammatory conditions	1,490 (3.4%)	925 (6.5%)	612 (7.2%)
Chronic Kidney Disease	370 (0.8%)	175 (1.2%)	101 (1.2%)
**Immunosuppressive Drugs**			
Steroids (long course)	305 (0.7%)	186 (1.3%)	114 (1.3%)
DMARDs	438 (1.0%)	283 (2.0%)	195 (2.3%)
MABs	154 (0.3%)	108 (0.8%)	68 (0.8%)

[1]n (%)

Table S2

Clinical Grouping	Conditions included
Ischaemic Heart Disease	coronary artery disease, angina, myocardial infarction
Neurological conditions	conditions affecting the brain or nerves, excluding stroke
Inflammatory conditions	rheumatoid arthritis, systemic lupus erythematosus, multiple sclerosis, ankylosing spondylitis, psoriasis/psoriatic arthropathy, coeliac disease, Crohn's disease, ulcerative colitis
Mental ill-health	depression, anxiety, schizophrenia, bipolar disorder, other psychiatric conditions
Severe respiratory disease	asthma or COPD requiring hospital admission or two or more courses of systemic steroids
Chronic viral infections	Hepatitis B, Hepatitis C, HIV
Haematological non-malignant conditions	thalassaemia, sickle cell disease, other inherited anaemias, non-malignant bone marrow disorders, primary and acquired immunodeficiencies, disorders/removal of the spleen
Haematological cancer	malignancies of the blood or bone marrow, active or past
Non-haematological cancer	solid organ tumours, active or past
Underweight	BMI<18.5
Overweight	BMI 25-29.9
Obesity	BMI 30+
Steroids - long course	Systemic steroid course lasting more than 14 days taken between November 2020-June 2021 (period over which first and second vaccine doses were rolled out)
Disease modifying anti-rheumatic drugs	azathioprine, cyclophosphamide, fingolimod, hydroxychloroquine, leflunomide, mercaptopurine, methotrexate, mycophenolate, sulfasalazine
Monoclonal antibody therapy	adalimumab, belimumab, canakinumab, certolizumab, golimumab, guselkumab, infliximab, ocrelizumab, rituximab, tocilizumab, ustekinumab, vedolizumab
Clinically Vulnerable	As per JCVI and UKHSA Green Book criteria: chronic respiratory disease, chronic heart disease and vascular disease, chronic kidney disease, chronic liver disease, chronic neurological disease including epilepsy, severe learning disability, diabetes, malignancy, morbid obesity, severe mental illness
Clinically Extremely Vulnerable	As per JCVI/NHS critieria: solid organ transplant recipients, cancer undergoing active chemotherapy or radical radiotherapy, cancer of blood or bone marrow, immunotherapy or antibody treatments for cancer, targeted cancer therapies affecting the immune system, bone marrow or stem cell transplant in last 6 months or still taking immunosuppressive drugs, severe respiratory conditions, immunosuppression, problems with spleen including splenectomy, Down's syndrome, severe chronic kidney disease, pregnancy with significant heart disease.

Table S3

Characteristic	Beta coefficient	Lower 95% CI	Upper 95% CI	P-value
Clinically vulnerable	-0.10	-0.16	-0.05	0.00
Clinically extremely vulnerable	-0.32	-0.38	-0.25	0.00
Ischaemic heart disease	-0.14	-0.25	-0.03	0.01
Severe respiratory disease	-0.04	-0.14	0.06	0.48
Solid Organ Cancer (Non-Haem)	0.01	-0.07	0.09	0.83
Haematological Cancer	-0.66	-0.89	-0.43	0.00
BMI - Underweight	-0.08	-0.31	0.15	0.51
BMI - Overweight	-0.01	-0.07	0.04	0.62
BMI - Obese	-0.13	-0.19	-0.06	0.00
Immunosuppression - Steroids (long course)	-0.55	-0.74	-0.36	0.00
Immunosuppression – DMARDs	-0.72	-0.87	-0.58	0.00
Immunosuppression - MABs	-1.42	-1.67	-1.17	0.00
Chronic viral (HBV, HCV, HIV)	0.18	-0.13	0.49	0.25
Type 1 Diabetes	-0.36	-0.66	-0.06	0.02
Type 2 Diabetes	-0.18	-0.28	-0.07	0.00
Stroke	-0.01	-0.19	0.17	0.88
Heart failure	-0.58	-0.90	-0.25	0.00
Chronic Kidney Disease	-0.51	-0.72	-0.31	0.00
Neurological condition (excl. stroke)	-0.11	-0.27	0.05	0.20
Asthma	-0.07	-0.13	-0.01	0.03
COPD	-0.08	-0.22	0.05	0.22
Hypertension	-0.07	-0.12	-0.02	0.01
Mental ill-health	-0.02	-0.20	0.17	0.87
Liver condition	-0.02	-0.19	0.16	0.85
Inflammatory condition	-0.22	-0.30	-0.13	0.00
Haematological non-malignant condition	-0.31	-0.59	-0.02	0.03

Figure S2

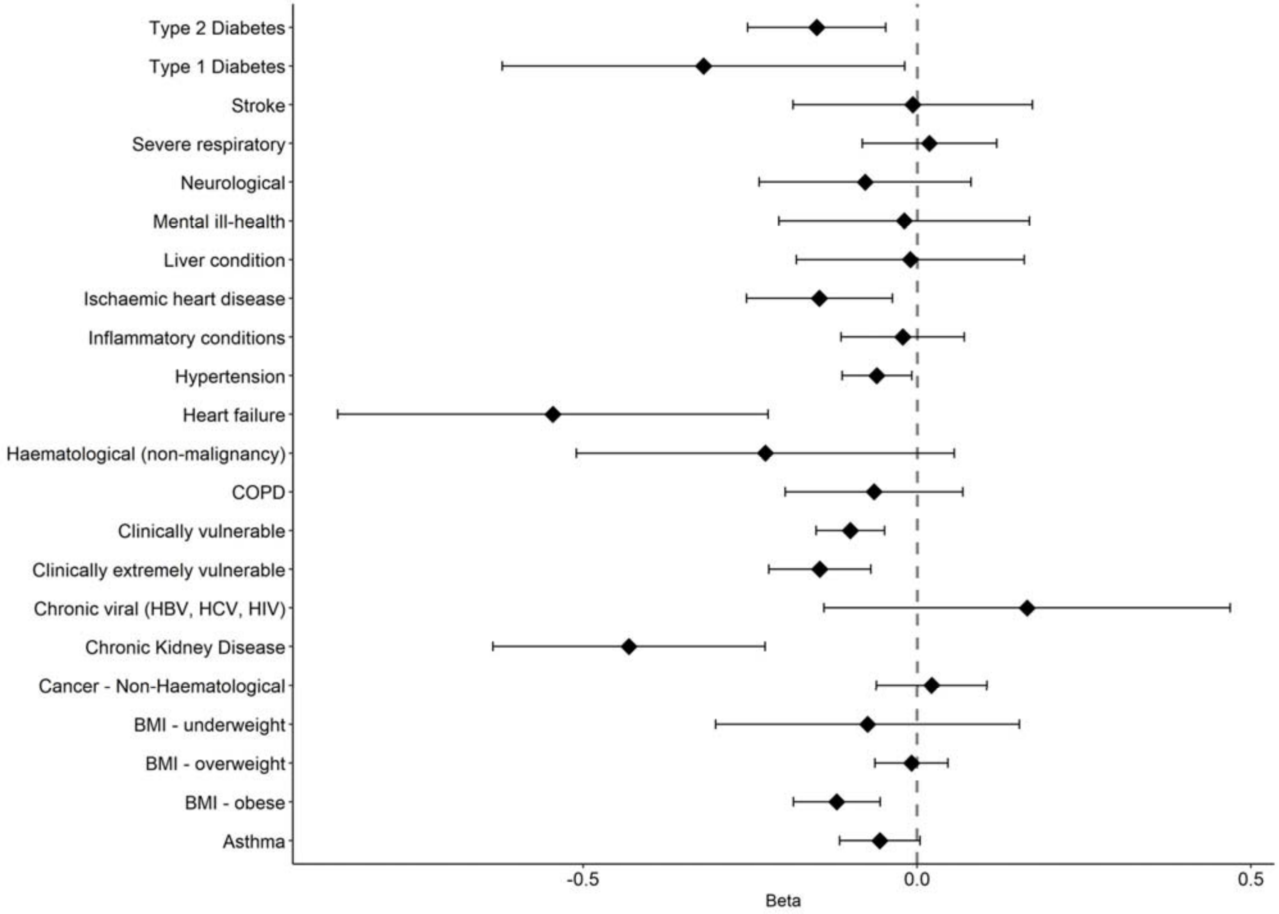
Type 2 Diabetes
Type 1 Diabetes
Stroke
Severe respiratory
Neurological
Mental ill-health
Liver condition
Ischaemic heart disease
Inflammatory conditions
Hypertension
Heart failure
Haematological (non-malignancy)
COPD
Clinically vulnerable
Clinically extremely vulnerable
Chronic viral (HBV, HCV, HIV)
Chronic Kidney Disease
Cancer - Non-Haematological
BMI - underweight
BMI - overweight
BMI - obese
Asthma
-0.5
0.0
0.5
Beta

Table S4

Characteristic	Beta coefficient	Lower 95% CI	Upper 95% CI	P-value
Clinically vulnerable	-0.10	-0.15	-0.05	0.00
Clinically extremely vulnerable	-0.15	-0.22	-0.07	0.00
Ischaemic heart disease	-0.15	-0.26	-0.04	0.01
Severe respiratory disease	0.02	-0.08	0.12	0.73
Cancer - Non-Haematological	0.02	-0.06	0.10	0.61
BMI - underweight	-0.07	-0.30	0.15	0.52
BMI - overweight	-0.01	-0.06	0.05	0.76
BMI - obese	-0.12	-0.19	-0.06	0.00
Chronic viral (HBV, HCV, HIV)	0.16	-0.14	0.47	0.29
Type 1 Diabetes	-0.32	-0.62	-0.02	0.04
Type 2 Diabetes	-0.15	-0.25	-0.05	0.00
Stroke	-0.01	-0.19	0.17	0.94
Heart failure	-0.55	-0.87	-0.22	0.00
Chronic Kidney Disease	-0.43	-0.64	-0.23	0.00
Neurological condition (excl. stroke)	-0.08	-0.24	0.08	0.33
Asthma	-0.06	-0.12	0.00	0.07
COPD	-0.07	-0.20	0.07	0.34
Hypertension	-0.06	-0.11	-0.01	0.02
Mental ill-health	-0.02	-0.21	0.17	0.84
Liver condition	-0.01	-0.18	0.16	0.90
Inflammatory condition	-0.02	-0.11	0.07	0.64
Haematological non-malignant condition	-0.23	-0.51	0.06	0.11
