# Supplementary material for: Spike-antibody responses to COVID-19 vaccination by demographic and clinical factors in a prospective community cohort study
Source: Nat Commun. 2022 Oct 2;13:5780. doi: 10.1038/s41467-022-33550-z (PMC9526787; doi:10.1038/s41467-022-33550-z)
Supplement: Supplementary file 2 — Reporting Summary [file 41467_2022_33550_MOESM2_ESM.pdf]

nature portfolio

Corresponding author(s): Prof R W Aldridge

Last updated by author(s): 11/09/2022

Reporting Summary

Nature Portfolio wishes to improve the reproducibility of the work that we publish. This form provides structure for consistency and transparency in reporting. For further information on Nature Portfolio policies, see our Editorial Policies and the Editorial Policy Checklist.

Statistics

For all statistical analyses, confirm that the following items are present in the figure legend, table legend, main text, or Methods section.

n/a	Confirmed	
☐	☒	The exact sample size (*n*) for each experimental group/condition, given as a discrete number and unit of measurement
☐	☒	A statement on whether measurements were taken from distinct samples or whether the same sample was measured repeatedly
☐	☒	The statistical test(s) used AND whether they are one- or two-sided<br>*Only common tests should be described solely by name; describe more complex techniques in the Methods section.*
☐	☒	A description of all covariates tested
☐	☒	A description of any assumptions or corrections, such as tests of normality and adjustment for multiple comparisons
☐	☒	A full description of the statistical parameters including central tendency (e.g. means) or other basic estimates (e.g. regression coefficient) AND variation (e.g. standard deviation) or associated estimates of uncertainty (e.g. confidence intervals)
☐	☒	For null hypothesis testing, the test statistic (e.g. *F*, *t*, *r*) with confidence intervals, effect sizes, degrees of freedom and *P* value noted<br>*Give P values as exact values whenever suitable.*
☒	☐	For Bayesian analysis, information on the choice of priors and Markov chain Monte Carlo settings
☒	☐	For hierarchical and complex designs, identification of the appropriate level for tests and full reporting of outcomes
☐	☒	Estimates of effect sizes (e.g. Cohen's *d*, Pearson's *r*), indicating how they were calculated

Our web collection on statistics for biologists contains articles on many of the points above.

Software and code

Policy information about availability of computer code

Data collection	Survey data were collected from participants using REDCap 12.4.0 https://www.project-redcap.org/. Routine vaccination data were obtained by data linkage through NHS Digital.
Data analysis	Data analyses were conducted in R R 4.0.3.

For manuscripts utilizing custom algorithms or software that are central to the research but not yet described in published literature, software must be made available to editors and reviewers. We strongly encourage code deposition in a community repository (e.g. GitHub). See the Nature Portfolio guidelines for submitting code & software for further information.

Data

Policy information about availability of data

All manuscripts must include a data availability statement. This statement should provide the following information, where applicable:

- Accession codes, unique identifiers, or web links for publicly available datasets
- A description of any restrictions on data availability
- For clinical datasets or third party data, please ensure that the statement adheres to our policy

Data used in this study are available from the Office of National Statistics Secure Research Service.

Field-specific reporting

Please select the one below that is the best fit for your research. If you are not sure, read the appropriate sections before making your selection.

[X] Life sciences [ ] Behavioural & social sciences [ ] Ecological, evolutionary & environmental sciences

For a reference copy of the document with all sections, see nature.com/documents/nr-reporting-summary-flat.pdf

Life sciences study design

All studies must disclose on these points even when the disclosure is negative.

Sample size	A-priori sample size calculations were carried out for the main Virus Watch cohort as reported in our study protocol http://dx.doi.org/10.1136/bmjopen-2020-048042.
Data exclusions	Exclusion criteria were pre-determined. Participants under the age of 18 were not eligible to submit serum samples. Participants with missing vaccination information or demographic information were excluded from all analyses. Samples that were positive for Nucleocapsid antibodies (indicating prior infection) were excluded from all analyses. Samples with invalid results for Nucleocapsid or Spike antibodies were excluded from all analyses. Following receipt of Dose 2, samples that were tested without the requisite 1:100 dilution were excluded from all analyses due to the likelihood of exceeding the upper limit of the assay. Samples taken on the day of or after receipt of Dose 3 were excluded from all analyses. Samples taken <28 days after Dose 2 were excluded from the Dose 2 regression analysis, due to the likelihood of not yet having achieved peak antibody levels.
Replication	These findings have not yet been replicated to our knowledge.
Randomization	Randomisation was not possible as this was an observational study design.
Blinding	Serological testing was outsourced to a commercial laboratory. Staff conducting serological testing held no demographic or clinical information regarding participants and were blind to the vaccination and infection status of the participants who submitted serum samples.

Reporting for specific materials, systems and methods

We require information from authors about some types of materials, experimental systems and methods used in many studies. Here, indicate whether each material, system or method listed is relevant to your study. If you are not sure if a list item applies to your research, read the appropriate section before selecting a response.

Materials & experimental systems

n/a	Involved in the study	
[X]	[ ]	Antibodies
[X]	[ ]	Eukaryotic cell lines
[X]	[ ]	Palaeontology and archaeology
[X]	[ ]	Animals and other organisms
[ ]	[X]	Human research participants
[X]	[ ]	Clinical data
[X]	[ ]	Dual use research of concern

Methods

n/a	Involved in the study	
[X]	[ ]	ChIP-seq
[X]	[ ]	Flow cytometry
[X]	[ ]	MRI-based neuroimaging

Human research participants

Policy information about studies involving human research participants

Population characteristics	The study population was drawn from a large community cohort of over 44,000 individuals living in England and Wales, enrolled in a longitudinal study of SARS-CoV2 infection, immunity, and behaviour (Virus Watch). 8,356 adults aged 18 years and over were included in the main linear regression analysis, of whom 58% were female, 0.7% were aged 18-24 years, 8.3% wre aged 25-44 years, 43% were aged 45-64 years, and 48% were aged 65 years and over; 92% of individuals reported their ethnicity as White British; 67% of included individuals received a primary course of Oxford-AstraZeneca's ChAdOx1-S vaccine, while 33% received Pfizer's BNT162b2 vaccine.
Recruitment	We used a range of methods including the Royal Mail Post Office Address File to generate a random list of residential address lists that were sent recruitment postcards, we placed social media adverts on Facebook and Twitter and sent SMS messages and letters to participants from their General Practitioners. Participants enrolled in the study on a whole-household basis (i.e. all individuals in a household had to consent to participation in order for the household to enrol). Adults aged 18 and over enrolled in the main Virus Watch study were invited to participate in monthly serum sampling from February 2021 onwards, in order to study antibody responses to vaccines. Invitations were sent in waves, starting with those who had already self-reported vaccination, or who were known to be in clinical groups that were nationally prioritised for vaccination as per their self-reported clinical information. A subset of the whole cohort gave written informed consent to participate in serum

sampling and provided samples after vaccination. It is possible that recruitment and consent methods were susceptible to healthy volunteer self-selection bias, however due to the large sample size, geographical reach, and household-based enrollment, we were able to achieve good representation across all ages and most important clinical conditions. Our findings may not be generalisable to some populations such as ethnic minority groups who were under-represented in the study, marginalised populations including individuals without a fixed abode who were not included in the study, frail or elderly populations who may not have been able to participate in the study, and those residing in long-term care facilities who were not included in the study. Furthermore, these findings are unlikely to be generalisable to children and young adults aged under 18 years who were excluded from serum sampling.

Ethics oversight

This study has been approved by the Hampstead NHS Health Research Authority Ethics Committee. Ethics approval number - 20/HRA/2320.

Note that full information on the approval of the study protocol must also be provided in the manuscript.
